# Association of a Perioperative Multicomponent Fall Prevention Intervention With Falls and Quality of Life After Elective Inpatient Surgical Procedures

**DOI:** 10.1001/jamanetworkopen.2022.1938

**Published:** 2022-03-11

**Authors:** Bradley A. Fritz, Christopher R. King, Divya Mehta, Emily Somerville, Alex Kronzer, Arbi Ben Abdallah, Troy Wildes, Michael S. Avidan, Eric J. Lenze, Susan Stark

**Affiliations:** 1 Department of Anesthesiology, Washington University School of Medicine in St Louis, St Louis, Missouri; 2 Program in Occupational Therapy, Washington University School of Medicine in St Louis, St Louis, Missouri; 3 Department of Psychiatry, Washington University School of Medicine in St Louis, St Louis, Missouri

## Abstract

**Importance:**

Falls after elective inpatient surgical procedures are common and have physical, emotional, and financial consequences. Close interactions between patients and health care teams before and after surgical procedures may offer opportunities to address modifiable risk factors associated with falls.

**Objective:**

To assess whether a multicomponent intervention that incorporates education, home medication review, and home safety assessment is associated with reductions in the incidence of falls after elective inpatient surgical procedures.

**Design, Setting, and Participants:**

This prospective propensity score–matched cohort study was a prespecified secondary analysis of data from the Electroencephalography Guidance of Anesthesia to Alleviate Geriatric Syndromes (ENGAGES) randomized clinical trial, which was conducted at a single academic medical center between January 16, 2015, and May 7, 2018. Patients in the intervention group of the present study were enrolled in either arm of the ENGAGES clinical trial. Patients in the control group were selected from the Systematic Assessment and Targeted Improvement of Services Following Yearly Surgical Outcomes Surveys prospective observational cohort study, which created a registry of patient-reported postoperative outcomes at the same single center. The propensity score–matched cohort in the present study included 1396 patients (698 pairs) selected from a pool of 2013 eligible patients. All patients underwent elective surgical procedures with general anesthesia and had a hospital stay of 2 or more days. Data were analyzed from January 2, 2020, to January 11, 2022.

**Interventions:**

The multicomponent safety intervention (offered to all patients in the ENGAGES clinical trial) included patient education on fall prevention techniques, home medication review by a geriatric psychiatrist (with communication of recommended changes to the surgeon), a self-administered home safety assessment, and targeted occupational therapy home visits with home hazard removal (offered to patients with a preoperative history of falls).

**Main Outcomes and Measures:**

The primary outcome was patient-reported falls within 1 year after an elective inpatient surgical procedure. The secondary outcome was quality of life 1 year after an elective surgical procedure, which was measured using the physical and mental composite summary scores on the Veterans RAND 12-item health survey (score range, 0-100 points, with 0 indicating lowest quality of life and 100 indicating highest quality of life).

**Results:**

Among 1396 patients, the median age was 69 years (IQR, 64-75 years), and 739 patients (52.9%) were male. With regard to race, 5 patients (0.4%) were Asian, 97 (6.9%) were Black or African American, 2 (0.1%) were Native Hawaiian or Pacific Islander, 1237 (88.6%) were White, 3 (0.2%) were of other race, and 52 (3.7%) were of unknown race; with regard to ethnicity, 12 patients (0.9%) were Hispanic or Latino, 1335 (95.6%) were non-Hispanic or non-Latino, and 49 (3.5%) were of unknown ethnicity. Adherence to individual intervention components was modest (from 22.9% for completion of the self-administered home safety assessment to 28.2% for implementation of the geriatric psychiatrist's recommended medication changes). Falls within 1 year after surgical procedures were reported by 228 of 698 patients (32.7%) in the intervention group and 225 of 698 patients (32.2%) in the control group. No significant difference was found in falls between the 2 groups (standardized risk difference, 0.4%; 95% CI, −4.5% to 5.3%). After adjusting for preoperative quality of life, patients in the intervention group had higher physical composite summary scores (3.8 points; 95% CI, 2.4-5.1 points) and higher mental composite summary scores (5.7 points; 95% CI, 4.7-6.7 points) at 1 year compared with patients in the control group.

**Conclusions and Relevance:**

In this cohort study, a multicomponent safety intervention was not associated with reductions in falls within the first year after an elective surgical procedure; however, an increase in quality of life at 1 year was observed. These results suggest a need for other interventions, such as those designed to increase adherence, to lower the incidence of falls after surgical procedures.

## Key Points

**Question:**

Is an intervention that incorporates patient education, home medication review, and hazard identification in the home environment associated with reductions in falls during the first year after an elective inpatient surgical procedure?

**Findings:**

In this cohort study involving 1396 patients (698 pairs) presenting for elective inpatient surgical procedures, 33% of patients in the intervention group and 32% of patients in the control group experienced a fall within 1 year after undergoing a surgical procedure, representing a nonsignificant difference.

**Meaning:**

These results suggest that other interventions may need to be developed to reduce postoperative fall incidence among patients receiving elective inpatient surgical procedures.

## Teaser

This cohort study assesses whether a multicomponent intervention incorporating education, home medication review, and home safety assessment is associated with reductions in the incidence of postoperative falls among patients receiving elective inpatient surgical procedures.

## Introduction

Falls after elective inpatient surgical procedures are a substantial concern, and this public health problem is becoming increasingly important as the population ages. Approximately 1% to 4% of patients fall while in the hospital after undergoing a surgical procedure.^1,2^ After discharge, patients experience falls at rates up to 3 times higher than other community-dwelling adults.^2^ These falls have serious consequences. In the US, more than 25 000 individuals 75 years or older died of fall-related injuries in 2016, and that number has increased consistently since the turn of the millennium.^3^ Falls lead to injuries ranging from minor cuts and bruises to head injuries, reductions in functional independence, and emotional impacts, such as ongoing fear of falling.^4,5^ Falls also have a substantial economic impact; each year, US insurers spend $30 billion to $50 billion on fall-related care.^6,7^ Increased fall risk after surgical procedures means surgical patients have greater potential to benefit from fall prevention interventions.

Because elective inpatient surgical procedures are carefully planned and followed up by a period of close interaction with the health care system, the preoperative and postoperative periods may be ideal times to apply fall prevention interventions. Effective fall prevention requires multicomponent interventions because fall risk is associated with many factors.^8^ Although some important risk factors, such as age^1,2,9^ and previous falls,^9,10,11^ are not modifiable, other well-established risk factors, such as polypharmacy (use of a large number of home medications)^11,12^ and hazards in the home environment,^13^ can be modified. Therefore, we hypothesized that a multicomponent fall prevention intervention focused on simplifying medication lists and removing hazards in the home would be associated with reductions in falls during the first year after an elective inpatient surgical procedure.

## Methods

### Study Design

This prospective propensity score–matched cohort study comprised a prespecified secondary analysis of data from the Electroencephalography Guidance of Anesthesia to Alleviate Geriatric Syndromes (ENGAGES) randomized clinical trial.^14,15^ The primary purpose of the single-center ENGAGES clinical trial was to determine the effect of an electroencephalography-guided anesthesia intervention on postoperative delirium. A secondary purpose of the clinical trial, explored in the current cohort study, was to assess whether a multicomponent safety intervention would be associated with reductions in the incidence of postoperative falls. The multicomponent safety intervention was offered to all patients in the ENGAGES clinical trial. Patients in the control group of the present study were selected from the Systematic Assessment and Targeted Improvement of Services Following Yearly Surgical Outcomes Surveys (SATISFY-SOS) prospective observational cohort study, which created a registry of patient-reported postoperative outcomes at the same single center.^16^ The Human Research Protection Office at Washington University School of Medicine in St Louis, Missouri, approved both the ENGAGES and SATISFY-SOS studies, and all patients provided written informed consent. All patients enrolled in the ENGAGES clinical trial were concurrently enrolled in the SATISFY-SOS cohort study; only SATISFY-SOS participants who were not concurrently enrolled in ENGAGES were considered as potential controls. Both the ENGAGES and SATISFY-SOS studies were registered at ClinicalTrials.gov (identification Nos.: NCT02241655 and NCT02032030). The current study followed the Strengthening the Reporting of Observational Studies in Epidemiology (STROBE) reporting guideline for cohort studies.^17^

### Patient Population

The intervention group included patients enrolled in either arm of the ENGAGES clinical trial. The ENGAGES study included adult patients 60 years or older who were undergoing major surgical procedures with general anesthesia and had an anticipated hospital stay of 2 or more days at Barnes-Jewish Hospital (St Louis, Missouri) between January 16, 2015, and May 7, 2018. Exclusion criteria included neurosurgical procedures, preoperative delirium, blindness, deafness, and inability to read or converse in the English language.

The control group was selected from the SATISFY-SOS study. The SATISFY-SOS included adult patients 18 years or older who underwent surgical procedures with anesthesia at Barnes-Jewish Hospital between July 1, 2012, and June 30, 2017 (with follow-up continued until 2018). Patients in the SATISFY-SOS study had health characteristics similar to those of the broader population of patients evaluated at our preoperative assessment clinic.^16^ To mimic the inclusion criteria used in the ENGAGES clinical trial, the control group was limited to SATISFY-SOS participants 60 years or older who underwent nonneurological surgical procedures between March 24, 2014, and June 30, 2017, had a hospital stay of 2 or more days, and completed a survey about postoperative falls 1 year after receiving a surgical procedure. In addition, patients who received orthopedic surgical procedures were excluded because these procedures were rare (n = 1) in the ENGAGES study.

### Multicomponent Safety Intervention

All patients enrolled in the ENGAGES clinical trial received a safety intervention containing up to 4 components. First, all patients received a copy of the Tailoring Interventions for Patient Safety fall information sheet for patients,^18^ which outlines strategies to avoid in-hospital falls. Second, a geriatric psychiatrist (E.J.L.) reviewed the home medication list for drugs or drug combinations (eg, benzodiazepine, centrally acting anticholinergic, or antihistamine medications) likely to increase the risk of falls or confusion after a surgical procedure. The psychiatrist communicated in writing with the patient’s surgeon to recommend potential changes to home medications at the time of hospital discharge. Third, each patient was asked to complete the Home Safety Self Assessment Tool (HSSAT), version 4,^19^ a self-administered checklist that guides patients to identify safety hazards in their home environment and suggests possible solutions for each hazard. Fourth, patients reporting a preoperative history of falls who lived fewer than 45 miles from the hospital were eligible to receive a home visit from an occupational therapist (including E.S. and S.S.). During the visit, the occupational therapist conducted the Westmead Home Safety Assessment^20^ to identify hazards in the home environment and provided home modifications (eg, motion sensor lights on staircases or tape to secure rugs) to remove hazards. A contractor installed any necessary architectural changes (eg, grab bars or handrails), and the occupational therapist trained patients on all implemented modifications. Patients enrolled in the SATISFY-SOS study (control group) did not receive any of these interventions.

### Outcome Measurement

The primary outcome was patient-reported falls within 1 year after an elective inpatient surgical procedure. Patients in the ENGAGES and SATISFY-SOS studies received surveys approximately 1 month postoperatively and 1 year postoperatively; these surveys included questions about falls occurring since the surgical procedure. Surveys were sent via email and/or postal mail, with phone calls made to nonresponders. A fall was defined as an unexpected event in which the patient landed on the floor, ground, or lower level, consistent with the Prevention of Falls Network Europe definition.^21^ The primary outcome was defined as a patient-reported fall on either postoperative survey. Most surveys (with the exception of an early version of the SATISFY-SOS 1-month survey) also asked whether falls led to injuries.

The secondary outcome was patient-reported quality of life at 1 year after the surgical procedure. In both the ENGAGES and SATISFY-SOS studies, quality of life was assessed using the physical composite summary (PCS-12) and mental composite summary (MCS-12) of the Veterans RAND 12-item health survey^22^ (score range 0-100, with 0 indicating lowest quality of life and 100 indicating highest quality of life), which was completed by participants before the surgical procedure, approximately 1 month after the procedure, and 1 year after the procedure. The US population mean (SD) score for each composite summary is 50 (10) points.^23^

### Statistical Analysis

All analyses were conducted from January 2, 2020, to January 11, 2022, using R software, version 4.0.3 (R Foundation for Statistical Computing); the R code used is available on GitHub.^24^ Statistical significance was defined as 2-sided *P* < .05. Patients in the intervention group were matched 1:1 with patients in the control group based on propensity scores. Propensity scores were calculated using a logistic regression model in which the outcome was enrollment in the ENGAGES clinical trial. Variables in the regression model included age, sex, American Society of Anesthesiologists physical status (class I-VI, with I indicating normal health, II indicating mild systemic disease, III indicating severe systemic disease, IV indicating severe systemic disease that is a constant threat to life, V indicating moribund and not expected to survive without an operation, and VI indicating brain death),^25^ history of falls, number of comorbid conditions, duration of anesthesia, and type of surgical procedure. Propensity score matching was performed using the MatchIt package for R software with a caliper of 0.2 SDs (selected post hoc based on convention).^26,27^ In addition, exact matches were required for American Society of Anesthesiologists physical status and duration of anesthesia greater than 5 hours because these features were considered to be important and did not balance in an initial match. A post hoc power calculation that assumed a baseline fall incidence of 25.0%^2^ indicated that a sample of at least 686 patients per group would be required to detect an absolute decrease in fall incidence of 6.25% (a relative decrease of 25.0%) with 80% power at α = .05.

To examine whether the matched cohorts were well balanced with respect to measured potential confounders, preoperative and intraoperative characteristics of the groups were compared using Pearson χ^2^ or Wilcoxon rank sum tests, as appropriate. The incidence of postoperative falls within 1 year was compared between the intervention and control groups in an intention-to-treat approach using a χ^2^ test and standardized risk difference. Median PCS-12 and MCS-12 scores at 1 year were compared between the groups using median regression analysis (quantreg package for R software), with the preoperative score included as an additional variable in the regression.

For exploratory analyses, 30-day outcomes and falls leading to injury were also compared between the groups using analogous methods. Because adherence to individual components of the safety intervention was variable, subgroup analyses were performed comparing falls at 1 year among ENGAGES participants who adhered to each intervention component (ie, medication changes were recommended, discharge medication list reflected the recommendations, HSSAT was completed, home environment was changed based on the HSSAT, and a home visit was performed by an occupational therapist) and matched participants in the control group.

## Results

**Figure 1.**
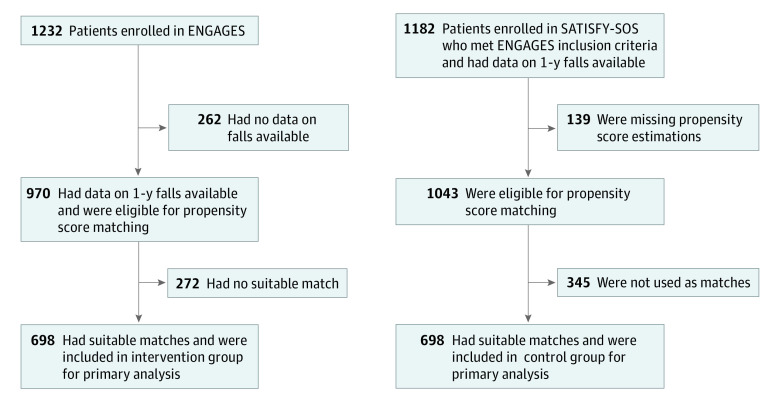
Study Flow Diagram ENGAGES indicates Electroencephalography Guidance of Anesthesia to Alleviate Geriatric Syndromes clinical trial; and SATISFY-SOS, Systematic Assessment and Targeted Improvement of Services Following Yearly Surgical Outcomes Surveys cohort study.

The propensity score–matched cohort included 1396 patients (698 pairs) selected from a pool of 2013 eligible patients (Figure 1). Before the matching process, propensity scores at enrollment in the ENGAGES clinical trial were inadequately balanced between the intervention group (from the ENGAGES study) and the control group (from the SATISFY-SOS study) (eFigure 1 in Supplement 1), which was a result of multiple differences in demographic characteristics, including sex, American Society of Anesthesiologists physical status, and types of surgical procedure received (eTable 1 in Supplement 1). After matching, propensity scores were well balanced between the groups (median pairwise difference in propensity scores, 0.016 [IQR, 0.001-0.028]) (eFigure 2 in Supplement 1). The total cohort had a median age of 69 years (IQR, 64-75 years) and included 739 men (52.9%) and 657 women (47.1%). With regard to race, 5 patients (0.4%) were Asian, 97 (6.9%) were Black or African American, 2 (0.1%) were Native Hawaiian or Pacific Islander, 1237 (88.6%) were White, 3 (0.2%) were of other race, and 52 (3.7%) were of unknown race; with regard to ethnicity, 12 patients (0.9%) were Hispanic or Latino, 1335 (95.6%) were non-Hispanic or non-Latino, and 49 (3.5%) were of unknown ethnicity. No significant differences were observed in preoperative or surgical characteristics between groups (eg, 433 patients [62.0%] in both groups had American Society of Anesthesiologists class III physical status; 215 patients [30.8%] in the intervention group vs 213 patients [30.5%] in the control group received cardiothoracic procedures) (Table 1).

**Table 1.** Preoperative and Surgical Characteristics of the Matched Cohorts

Characteristics	No./total No. (%)		*P* value
	Intervention cohort	Control cohort	
Total participants, No.	698	698	NA
Age, median (range), y	69 (64-74)	69 (64-75)	.91
Sex			
Female	319/698 (45.7)	338/698 (48.4)	.33
Male	379/698 (54.3)	360/698 (51.6)	
Race			
Asian	3/698 (0.4)	2/698 (0.3)	.07^a^
Black or African American	57/698 (8.2)	40/698 (5.7)	
Native Hawaiian or Pacific Islander	2/698 (0.3)	0	
White	633/698 (90.7)	604/698 (86.5)	
Other	3/698 (0.4)	0	
Unknown	0	52/698 (7.4)	
Ethnicity			
Hispanic or Latino	8/698 (1.1)	4/698 (0.6)	.44^b^
Non-Hispanic or non-Latino	686/698 (98.3)	649/698 (93.0)	
Unknown	4/698 (0.6)	45/698 (6.4)	
ASA physical status^c^			
I	1/698 (0.1)	1/698 (0.1)	NA^d^
II	141/698 (20.2)	141/698 (20.2)	
III	433/698 (62.0)	433/698 (62.0)	
IV	123/698 (17.6)	123/698 (17.6)	
No. of comorbid conditions, median (range)	4 (3-6)	4 (2-6)	.11
Preoperative SBT score, median (range)^e^	2 (0-4)	2 (0-4)	.62
Preoperative MET score for functional capacity^f^			
<4	289/679 (42.6)	276/682 (40.5)	.27
4-6	369/679 (54.3)	385/682 (56.5)	
>6-10	12/679 (1.8)	18/682 (2.6)	
Unable to assess	9 (1.3)	3/682 (0.4)	
History of falls	138/698 (19.8)	149/698 (21.3)	.51
Preoperative PCS-12 score, median (IQR)^g^	39.7 (30.4-49.4)	41.2 (31.9-50.8)	.09
Preoperative MCS-12 score, median (IQR)^g^	57.6 (49.5-61.9)	56.9 (47.1-61.1)	.06
Type of surgical procedure			
Cardiothoracic	215/698 (30.8)	213/698 (30.5)	.69
Gastrointestinal	119/698 (17.0)	126/698 (18.1)	
Gynecologic	81/698 (11.6)	68/698 (9.7)	
Hepatobiliary	87/698 (12.5)	75/698 (10.7)	
Urologic	61/698 (8.7)	62/698 (8.9)	
Vascular	64/698 (9.2)	67/698 (9.6)	
Other	71/698 (10.2)	87/698 (12.5)	
Duration of anesthesia, median (IQR), min	275 (208-355)	259 (195-361)	.13

Abbreviations: ASA, American Society of Anesthesiologists; MCS-12, mental composite summary on the Veterans RAND 12-item health survey; MET, metabolic equivalent; NA, not applicable; PCS-12, physical composite summary on the Veterans RAND 12-item health survey; SBT, Short Blessed Test.

^a^ When performing the χ^2^ test for this *P* value, Asian, Native Hawaiian or Pacific Islander, and other races were grouped together as a single category. Unknown race was treated as missing data.

^b^ When performing the χ^2^ test for this *P* value, unknown ethnicity was treated as missing data.

^c^ ASA physical status ranges from I to VI, with I indicating normal health, II indicating mild systemic disease, III indicating severe systemic disease, IV indicating severe systemic disease that is a constant threat to life, V indicating moribund and not expected to survive without an operation, and VI indicating brain death.

^d^ No statistical comparison was conducted for this variable because exact matching was performed.

^e^ Score range, 0 to 28, with 0 to 4 indicating normal cognitive status, 5 to 9 indicating questionable cognitive impairment, and 10 or more indicating impairment consistent with dementia.

^f^ Functional capacity MET scores range from 0 to 10, with less than 4 indicating capacity for low-intensity physical activity, 4 to 6 indicating capacity for moderate-intensity physical activity, and more than 6 to 10 indicating capacity for high-intensity physical activity.

^g^ Score range, 0 to 100, with 0 indicating lowest quality of life and 100 indicating highest quality of life.

### Intervention

All patients in the intervention group received the preoperative educational materials. The geriatric psychiatrist reviewed the home medication list for all 698 patients and recommended changes for 213 of those patients (30.5%), with discontinuation of medication the most common change (188 of 213 patients [88.3%]). Additional details of these recommendations are shown in eTable 2 in Supplement 1. The postoperative discharge medication list reflected the recommended changes among 60 of 213 patients (28.2%). Although all patients were given the HSSAT to complete at home, only 160 patients (22.9%) reported that they completed the self-assessment. Overall, 89 of 160 patients (55.6%) made changes to their home environment based on their self-assessment. Fifteen of 698 patients (2.1%) received a home visit from an occupational therapist.

### Outcomes in Intervention and Control Groups

In the primary intention-to-treat analysis, 228 of 698 patients in the intervention group (32.7%) and 225 of 698 patients in the control group (32.2%) reported a fall within the first year after their surgical procedure. There was no significant difference in the incidence of falls at 1 year between the 2 groups (difference, 0.4%; 95% CI, −4.5% to 5.3%). No significant differences were observed in falls at 30 days (difference, 1.6%; 95% CI, −1.7% to 4.9%) or in falls leading to injury at either 30 days (difference, 1.3%; 95% CI, −1.6% to 4.1%) or 1 year (difference, 4.0%; 95% CI, −0.1% to 8.2%) (Table 2). In subgroup analyses, no significant differences were found in falls at 1 year between matched patients in the control group and patients for whom medication changes were recommended (difference, 7.0%; 95% CI, −2.1% to 16.2%), patients with discharge medication lists that reflected the recommendations (difference, 5.0%; 95% CI, −12.3% to 22.3%), patients who completed the HSSAT (difference, −1.3%; 95% CI, −10.8% to 8.3%), patients who changed their home environment based on the HSSAT (difference, 1.1%; 95% CI, −12.7% to 15.0%), or patients who received a home visit from an occupational therapist (difference, 13.3%; 95% CI, −20.1% to 46.7%) (Table 3).

**Table 2.** Outcome Measures in the Matched Cohorts

Outcome	No./total No. (%)		Difference, % (95% CI)^a^	*P* value
	Intervention cohort (n = 698)	Control cohort (n = 698)		
Primary				
Falls at 1 y	228/698 (32.7)	225/698 (32.2)	0.4 (−4.5 to 5.3)	.86
Exploratory				
Falls with injury at 1 y	145/689 (21.0)	118/693 (17.0)	4.0 (−0.1 to 8.2)	.06
Falls at 30 d	66/651 (10.1)	46/537 (8.6)	1.6 (−1.7 to 4.9)	.36
Fall with injury at 30 d	33/651 (5.1)	10/265 (3.8)	1.3 (−1.6 to 4.1)	.40
Quality of life, median (IQR)				
PCS-12 score				
At 30 d	36.7 (28.7 to 43.8)	37.9 (31.1 to 45.4)	−0.7 (−2.1 to 0.8)	.39
At 1 y	43.6 (32.3 to 52.0)	40.7 (31.8 to 50.5)	3.8 (2.4 to 5.1)	<.001
MCS-12 score				
At 30 d	57.0 (48.8 to 61.7)	50.5 (44.0 to 54.8)	6.7 (5.8 to 7.6)	<.001
At 1 y	58.4 (51.6 to 62.1)	52.0 (45.4 to 59.1)	5.7 (4.7 to 6.7)	<.001

Abbreviations: MCS-12, mental composite summary on the Veterans RAND 12-item health survey; PCS-12, physical composite summary on the Veterans RAND 12-item health survey.

^a^ Differences in quality of life outcomes are coefficients from the quantile regression analysis, adjusted for preoperative PCS-12 scores when estimating postoperative PCS-12 scores and adjusted for preoperative MCS-12 scores when estimating postoperative MCS-12 scores. Full regression coefficients are shown in eTable 3 in Supplement 1.

**Table 3.** Incidence of Falls in Subgroup Analyses^a^

Subgroup	No. of pairs	Incidence of falls at 1 y, No. (%)		Difference, % (95% CI)	*P* value
		Intervention cohort	Control cohort		
Medication changes recommended	213	85 (39.9)	70 (32.9)	7.0 (−2.1 to 16.2)	.13
Discharge medication list reflected recommendations	60	24 (40.0)	21 (35.0)	5.0 (−12.3 to 22.3)	.57
HSSAT completed	160	40 (25.0)	42 (26.3)	−1.3 (−10.8 to 8.3)	.80
Patient changed home environment based on HSSAT	89	30 (33.7)	29 (32.6)	1.1 (−12.7 to 15.0)	.87
Received home visit from occupational therapist	15	6 (40.0)	4 (26.7)	13.3 (−20.1 to 46.7)	.44

Abbreviation: HSSAT, Home Safety Self Assessment Tool.

^a^ Each subgroup analysis includes participants in the Electroencephalography Guidance of Anesthesia to Alleviate Geriatric Syndromes clinical trial who adhered to a particular component of the multicomponent intervention along with matched participants in the control group.

**Figure 2.**
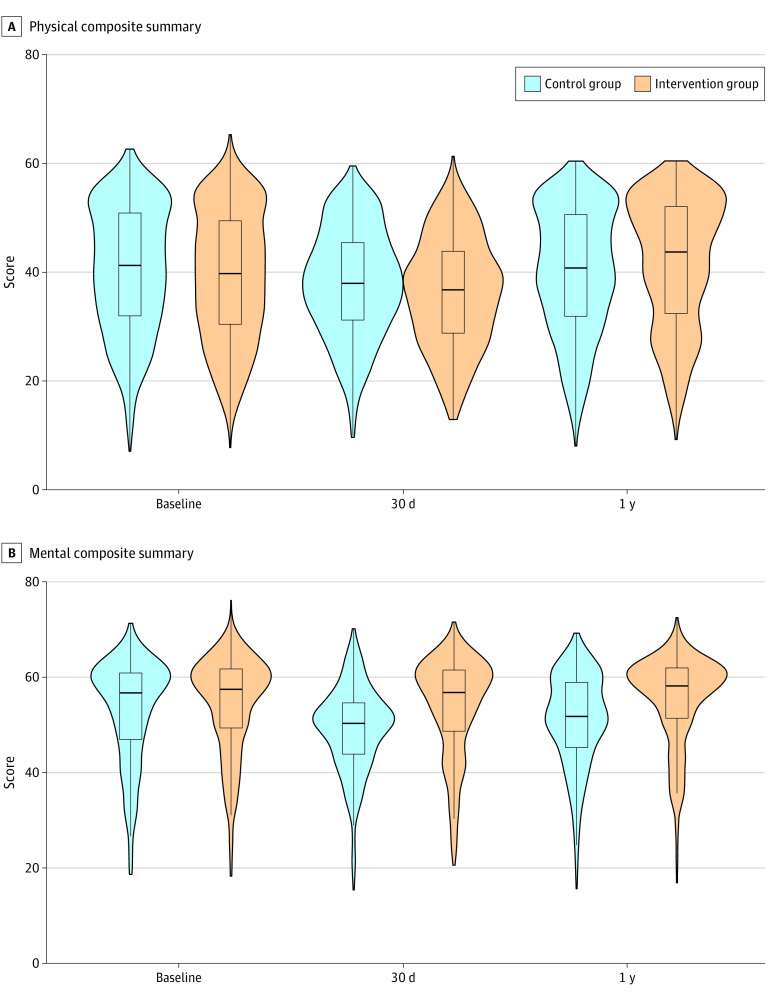
Quality of Life Patterns in the Intervention and Control Groups Violin and box plots showing the distribution of summary scores from Veterans RAND 12-item health surveys completed preoperatively, 30 days postoperatively, and 1 year postoperatively. In each box plot, the horizontal line shows the median score, box edges show 25th and 75th percentiles, and whiskers extend to the maximum and minimum values (excluding data points with a distance from the box edge >1.5 times the IQR).

The median PCS-12 score was 43.6 points (IQR, 32.3-52.0 points) at 1 year vs 39.7 points (IQR, 30.4-49.4 points) at baseline in the intervention group and 40.7 points (IQR, 31.8-50.5 points) at 1 year vs 41.2 points (IQR, 31.9-50.8 points) at baseline in the control group. After adjusting for preoperative PCS-12 scores, patients in the intervention group had higher 1-year postoperative PCS-12 scores than patients in the control group (difference, 3.8 points; 95% CI, 2.4-5.1 points) (model coefficients are shown in eTable 3 in Supplement 1). Both groups experienced decreases in PCS-12 scores at 1 month after their surgical procedure (intervention group: median, 36.7 points [IQR, 28.7-43.8 points]; control group: median, 37.9 points [IQR 31.1-45.4 points]), with a difference of −0.7 points (95% CI, −2.1 to 0.8 points) between groups compared with baseline (Figure 2).

The median MCS-12 score was 58.4 points (IQR, 51.6-62.1 points) at 1 year vs 57.6 points (IQR, 49.5-61.9 points) at baseline in the intervention group and 52.0 points (IQR, 45.4-59.1 points) at 1 year vs 56.9 points (IQR, 47.1-61.1 points) at baseline in the control group. After adjusting for preoperative MCS-12 scores, patients in the intervention group had higher 1-year postoperative MCS-12 scores than patients in the control group (difference, 5.7 points; 95% CI, 4.7-6.7 points). The median MCS-12 score was 57.0 points (IQR, 48.8-61.7 points) at 30 days in the intervention group and 50.5 points (IQR, 44.0-54.8 points) in the control group (adjusted difference, 6.7 points [95% CI, 5.8-7.6 points] between groups). Both groups experienced decreases in MCS-12 scores during the first month after their surgical procedure followed by small increases in both groups (Figure 2).

## Discussion

In this prospective propensity score–matched cohort study, a multicomponent fall prevention intervention was not associated with a change in fall incidence during the first year after a major elective surgical procedure. This result remained unchanged in subgroup analyses examining patients confirmed to receive each component of the intervention. In analyses of secondary outcomes, patients in the intervention group had better quality of life at 1 year after a surgical procedure compared with matched patients in the control group, as measured by both the PCS-12 and MCS-12.

Our fall prevention intervention used a pragmatic design that was intended to mimic real-world conditions in which uptake by patients may be incomplete. For example, only 22.9% of patients reported completing the self-guided home safety assessment. Reasons for low uptake are unknown, but it is plausible that patients received information on many topics preoperatively and did not prioritize the home safety assessment. In addition, only 28.2% of the medication changes recommended by the geriatric psychiatrist were reflected in discharge medication lists. It is unknown whether the lack of medication changes among the remaining patients were a result of disagreement with the psychiatrist’s recommendation, lack of consideration of the recommendation, or changes in patient status. Furthermore, it is unknown whether some of the discontinued medications were later resumed or whether newly prescribed medications were associated with increases in fall risk. Incomplete intervention uptake was the motivation for the performance of subgroup analyses involving patients who confirmed completion of each intervention. Although some subgroup analyses were limited by small samples, the lack of difference between groups in any of these analyses makes it less likely that incomplete intervention adherence was the sole explanation for the primary analysis results.

These results were inconsistent with those of a Cochrane meta-analysis^8^ reporting moderate-quality evidence that suggested multicomponent interventions were associated with reduced risk of falls in randomized clinical trials of community-dwelling older adults. The most common interventions reported were combinations of exercise with either home safety assessment or education. The meta-analysis^8^ results may differ from those of the present study because of differences in the interventions applied or differences in the patient population. However, our results were similar to those of 2 recently published large cluster-randomized clinical trials.^28,29^ The Prevention of Falls Injury Trial^28^ randomized patients 70 years or older to receive advice by mail, advice by mail plus a targeted exercise intervention, or advice by mail plus a targeted multifactorial intervention. The Strategies to Reduce Injuries and Develop Confidence in Elders clinical trial^29^ randomized patients 70 years or older to receive usual care or a multifactorial intervention comprising standardized risk assessment followed by individualized optimization of risk factors. Neither clinical trial found a difference in serious fall injuries between groups. The present study differed from both of the previous studies^28,29^ because it included surgical rather than medical patients and implemented a multicomponent intervention (in which all patients received the same set of interventions) rather than a multifactorial intervention (in which each patient received one of a subset of the available interventions based on individual risk assessment).

Caution is warranted when interpreting the secondary finding of improved quality of life in the intervention group compared with the control group. Given that the intervention was not associated with reductions in postoperative falls, the pretest probability for finding an association between the intervention and improved quality of life was low. The intervention may have been associated with reductions in other postoperative complications or adverse drug effects, which could have in turn mediated improved quality of life. Reliable information about postoperative complications among both groups was not available to allow further exploration. Although the groups were well balanced with regard to preoperative characteristics (Table 1), there may have been unmeasured confounders that explained the observed association; enrollment in a clinical trial may also have had beneficial consequences. Furthermore, the difference in PCS-12 scores between the groups was small, although the observed difference in MCS-12 scores was large enough to be clinically meaningful. In comparison, the Strategies to Reduce Injuries and Develop Confidence in Elders clinical trial^30^ did not find a meaningful impact of a fall prevention intervention on quality of life.

### Strengths and Limitations

This study has several strengths. First, the intervention was implemented by a multidisciplinary team that included anesthesiologists, geriatric psychiatrists, and occupational therapists. Each specialist group brought distinct expertise that maximized the likelihood of individual intervention components succeeding. In particular, home assessment interventions have previously been found to reduce falls when led by occupational therapists but not by other health care professionals,^31^ making the involvement of occupational therapists essential. Second, this study focused on outcomes that are of importance to patients. Third, a rigorous definition of falls was applied to permit these findings to be compared with those of other studies.

This study also has limitations. First, patients were questioned about falls infrequently (2 time points during the first year), so some falls may not have been reported accurately. The fall prevention intervention may have allowed patients in the intervention group to remember their falls more accurately, producing differential recall bias between the 2 groups. However, the overall fall rate is similar to the rate found in a review of previous studies,^2^ making it unlikely that a large number of falls were missed. Second, there is a risk that unmeasured confounders may be differentially distributed between the 2 study groups, even though the groups were well balanced with regard to measured confounders. In particular, the intervention group included patients who enrolled in a randomized clinical trial, whereas the control group was taken from a less selective prospective observational cohort study. If anything, selection of more engaged patients for enrollment in the randomized clinical trial would have biased the primary analysis away from the null hypothesis, making the lack of difference between the groups even more notable.

Third, the extent of adoption of several components of the intervention was modest, and increased adherence could have yielded different results. Although the subgroup analyses do not provide data to support that notion, these analyses were limited by the small sample. Fourth, the intervention focused on a limited number of risk factors. The addition of components, such as an exercise program, could have changed the results. Exercise programs may be particularly useful after surgical procedures, when temporary balance impairments exacerbate fall risk.^32^ Fifth, generalizability may be limited because of the single-center design and the nonrandom exclusion of healthier patients in the control group during the matching process.

## Conclusions

This prospective propensity score–matched cohort study found a multicomponent fall prevention intervention that included patient education, medication review, and home hazard assessment was not associated with any change in fall incidence during the first year after a major elective surgical procedure. Patients receiving the intervention reported better quality of life in both physical and mental domains compared with the control group, although the reason for those findings was unclear. Future studies examining fall interventions among postoperative patients might consider techniques to improve adherence to interventions, perhaps including risk assessment in a larger number of domains followed by personalized targeted interventions.

## Notes

Supplement 1. **eTable 1.** Preoperative and Surgical Characteristics of All Patients Meeting Eligibility Criteria **eTable 2.** Preoperative Home Medications, Changes Recommended by the Geriatric Psychiatrist, and Postoperative Discharge Medications in the Intervention Group **eTable 3.** Quantile Regression Coefficients **eFigure 1.** Distribution of Propensity Scores Among All Patients Meeting Eligibility Criteria **eFigure 2.** Distribution of Propensity Scores Among Matched Patients

Supplement 2.
Nonauthor Collaborators
