# Supplementary material for: Association of a Perioperative Multicomponent Fall Prevention Intervention With Falls and Quality of Life After Elective Inpatient Surgical Procedures
Source: JAMA Netw Open. 2022 Mar 11;5(3):e221938. doi: 10.1001/jamanetworkopen.2022.1938 (PMC8917421; doi:10.1001/jamanetworkopen.2022.1938)
Supplement: Supplement 1. — eTable 1. Preoperative and Surgical Characteristics of All Patients Meeting Eligibility Criteria eTable 2. Preoperative Home Medications, Changes Recommended by the Geriatric Psychiatrist, and Postoperative Discharge Medications in the Intervention Group eTable 3. Quantile Regression Coefficients eFigure 1. Distribution of Propensity Scores Among All Patients Meeting Eligibility Criteria eFigure 2. Distribution of Propensity Scores Among Matched Patients [file jamanetwopen-e221938-s001.pdf]

Supplementary Online Content

Fritz BA, King CR, Mehta D, et al; ENGAGES Research Group. Association of a perioperative multicomponent fall prevention intervention with falls and quality of life after elective inpatient surgical procedures. *JAMA Netw Open*. 2022;5(3):e221938. doi:10.1001/jamanetworkopen.2022.1938

eTable 1. Preoperative and Surgical Characteristics of All Patients Meeting Eligibility Criteria

eTable 2. Preoperative Home Medications, Changes Recommended by the Geriatric Psychiatrist, and Postoperative Discharge Medications in the Intervention Group

eTable 3. Quantile Regression Coefficients

eFigure 1. Distribution of Propensity Scores Among All Patients Meeting Eligibility Criteria

eFigure 2. Distribution of Propensity Scores Among Matched Patients

This supplementary material has been provided by the authors to give readers additional information about their work.

© 2022 Fritz BA et al. *JAMA Network Open*.

eTable 1. Preoperative and Surgical Characteristics of All Patients Meeting Eligibility Criteria (Matched and Unmatched; N = 2013)

**Variable**	**Intervention Cohort (N = 970)**	**Control Cohort (N = 1,043)**	**P**
Age	69 [64 to 74]	68 [64 to 74]	0.34
Sex			
Female	435 (45%)	541 (52%)	0.002
Male	535 (55%)	502 (48%)	
Race			
American Indian or Alaska Native	0 (0%)	1 (< 1%)	0.15
Asian	3 (< 1%)	3 (< 1%)	
Black or African American	82 (8%)	66 (6%)	
Native Hawaiian or Pacific Islander	2 (< 1%)	0 (0%)	
White	879 (91%)	897 (86%)	
Other	4 (< 1%)	0 (0%)	
Unknown	0 (0%)	76 (7%)	
Ethnicity			
Hispanic or Latino	9 (1%)	7 (1%)	0.78
Not Hispanic or Latino	957 (99%)	968 (93%)	
Unknown	4 (< 1%)	68 (6%)	
ASA Physical Status			
1	6 (1%)	2 (< 1%)	< 0.001
2	150 (15%)	303 (29%)	
3	487 (50%)	597 (57%)	
4	327 (34%)	140 (13%)	
5	0 (0%)	1 (< 1%)	
Number of Comorbid Conditions	4 [3 to 6]	3 [2 to 5]	< 0.001
Preoperative Short Blessed Test	2 [0 to 4]	2 [0 to 4]	0.70
Preoperative functional capacity			
6-10 METs	18 (2%)	30 (3%)	< 0.001
4-6 METs	468 (50%)	601 (59%)	
< 4 METs	435 (47%)	385 (38%)	
Unable to assess	11 (1%)	4 (< 1%)	
History of Falls	205 (21%)	236 (23%)	0.45
Preoperative PCS-12	38.5 [29.3 to 47.9]	41.4 [32.2 to 50.8]	< 0.001
Preoperative MCS-12	57.5 [48.7 to 61.7]	56.9 [47.5 to 61]	0.11
Surgical Service			
Cardiothoracic	426 (44%)	227 (22%)	< 0.001
Gastrointestinal	122 (12%)	249 (24%)	
Gynecologic	84 (9%)	94 (9%)	
Hepatobiliary	103 (11%)	85 (8%)	
Urology	72 (7%)	112 (11%)	
Vascular	86 (9%)	76 (7%)	
Other	77 (8%)	200 (19%)	
Duration of Anesthesia (min)	310 [230 to 396]	245 [181 to 338]	< 0.001

Abbreviations: ASA = American Society of Anesthesiologists. METs = metabolic equivalents. PCS-12 = physical composite summary. MCS-12 = mental composite summary

© 2022 Fritz BA et al. *JAMA Network Open*.

eTable 2. Preoperative Home Medications, Changes Recommended by the Geriatric Psychiatrist, and Postoperative Discharge Medications in the Intervention Group (n = 698)

Medication Class	Number of Patients Taking Medication Preoperatively	Number of Patients with Change Recommendations						Number of Patients Prescribed Medication at Hospital Discharge
		Any Rec [a]	Discontinue Medication	Reduce Dose	Replace Medication	Change Schedule	Other Rec	
Any Medication	690 (99%)	213	188	34	46	2	7	697 (100%)
ACE inhibitor	217 (31%)	0	0	0	0	0	0	162 (23%)
Acetylcholinesterase inhibitor for dementia	3 (0%)	0	0	0	0	0	0	2 (0%)
Acetylcholinesterase inhibitor for myasthenia gravis	0 (0%)	0	0	0	0	0	0	1 (0%)
Alpha-1 blocker	75 (11%)	1	1	1	0	0	0	111 (16%)
Alpha-2 agonist	18 (3%)	0	0	0	0	0	0	14 (2%)
Amphetamines	1 (0%)	0	0	0	0	0	0	0 (0%)
Analgesic- non-opioid	538 (77%)	16	15	1	0	0	0	662 (95%)
Analgesic- opioid	155 (22%)	4	3	3	1	0	1	600 (86%)
Angiotensin II receptor blocker	126 (18%)	1	1	0	0	0	0	87 (12%)
Anti-anxiety-other	4 (1%)	1	1	0	0	0	0	6 (1%)
Anti-cholinergic antispasmodics	48 (7%)	16	16	0	5	0	0	44 (6%)
Antidepressant-Other	28 (4%)	2	2	1	1	0	1	23 (3%)
Anti-parkinson medications	23 (3%)	0	0	0	0	0	0	20 (3%)
Antiarrhythmic	29 (4%)	0	0	0	0	0	0	63 (9%)
Anticoagulant	92 (13%)	0	0	0	0	0	0	253 (36%)
Anticonvulsant	86 (12%)	0	0	0	0	0	0	133 (19%)
Antiplatelet agent	70 (10%)	0	0	0	0	0	0	78 (11%)
Antipsychotics	25 (4%)	10	10	1	0	0	0	32 (5%)
Benzodiazepines	100 (14%)	78	65	25	2	1	2	106 (15%)
Beta blocker	304 (44%)	0	0	0	0	0	0	351 (50%)
Calcium channel blocker	199 (29%)	0	0	0	0	0	0	182 (26%)
Diuretic	260 (37%)	1	1	0	0	0	0	307 (44%)

© 2022 Fritz BA et al. *JAMA Network Open*.

H1-antihistamine	132 (19%)	65	60	0	8	0	3	114 (16%)
H2-antihistamine	62 (9%)	43	33	0	28	0	0	95 (14%)
Hypoglycemics	174 (25%)	0	0	0	0	0	0	177 (25%)
Immunosuppressant	61 (9%)	0	0	0	0	0	0	68 (10%)
Monoamine oxygenase inhibitors (MAOI)	0 (0%)	0	0	0	0	0	0	0 (0%)
Muscle relaxants	33 (5%)	16	14	2	1	0	2	41 (6%)
Nitrates	62 (9%)	0	0	0	0	0	0	42 (6%)
NMDA blocker for dementia	0 (0%)	0	0	0	0	0	0	0 (0%)
Nonbenzodiazepine hypnotic	21 (3%)	12	9	7	0	0	0	19 (3%)
Serotonergic antidepressants	147 (21%)	3	2	0	0	1	0	150 (21%)
Statin	366 (52%)	0	0	0	0	0	0	396 (57%)
Tricyclic antidepressants	25 (4%)	17	15	0	2	0	1	22 (3%)
Vasodilators-Other	52 (7%)	0	0	0	0	0	0	35 (5%)
Other	643 (92%)	15	14	0	2	0	0	690 (99%)
Medications per Patient, *median [interquartile range]*	10 [6-14]							13 [10-17]

Abbreviation: Rec = recommendation

a – The number in this column may be different from the sum of the following five columns because some patients had more than one recommendation for the same medication, or they had recommendations for more than one medication in the same class.

© 2022 Fritz BA et al. *JAMA Network Open*.

eTable 3. Quantile Regression Coefficients

Outcome	Predictor	Coefficient	Standard Error	P
PCS-12 at 30 days	Intercept	22.35	1.43	< 0.001
	Enroll in ENGAGES	-0.65	0.75	0.38
	Preoperative PCS-12	0.37	0.03	< 0.001
MCS-12 at 30 days	Intercept	28.90	1.96	< 0.001
	Enroll in ENGAGES	6.66	0.45	< 0.001
	Preoperative MCS-12	0.38	0.03	< 0.001
PCS-12 at 1 year	Intercept	13.41	1.42	< 0.001
	Enroll in ENGAGES	3.75	0.69	< 0.001
	Preoperative PCS-12	0.66	0.03	< 0.001
MCS-12 at 1 year	Intercept	33.14	1.46	< 0.001
	Enroll in ENGAGES	5.68	0.50	< 0.001
	Preoperative MCS-12	0.35	0.03	< 0.001

© 2022 Fritz BA et al. *JAMA Network Open*.

eFigure 1. Distribution of Propensity Scores Among All Patients Meeting Eligibility Criteria (Matched and Unmatched; N = 2013)

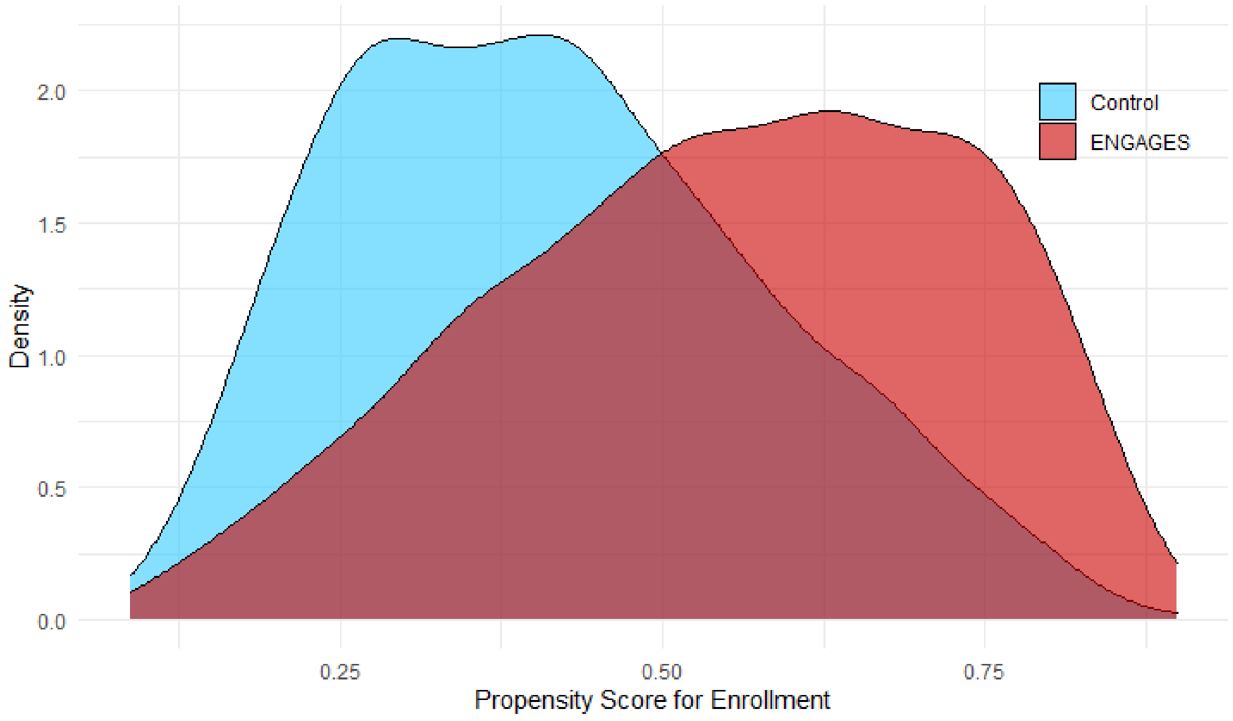

Density plot of propensity score for enrollment in the ENGAGES trial among all patients in the control (N = 1,043) and intervention groups (N = 970) who met the ENGAGES inclusion criteria, had one-year falls data available, and had no missing data for any of the variables in the propensity model.

© 2022 Fritz BA et al. *JAMA Network Open*.

eFigure 2. Distribution of Propensity Scores Among Matched Patients (n = 1396)

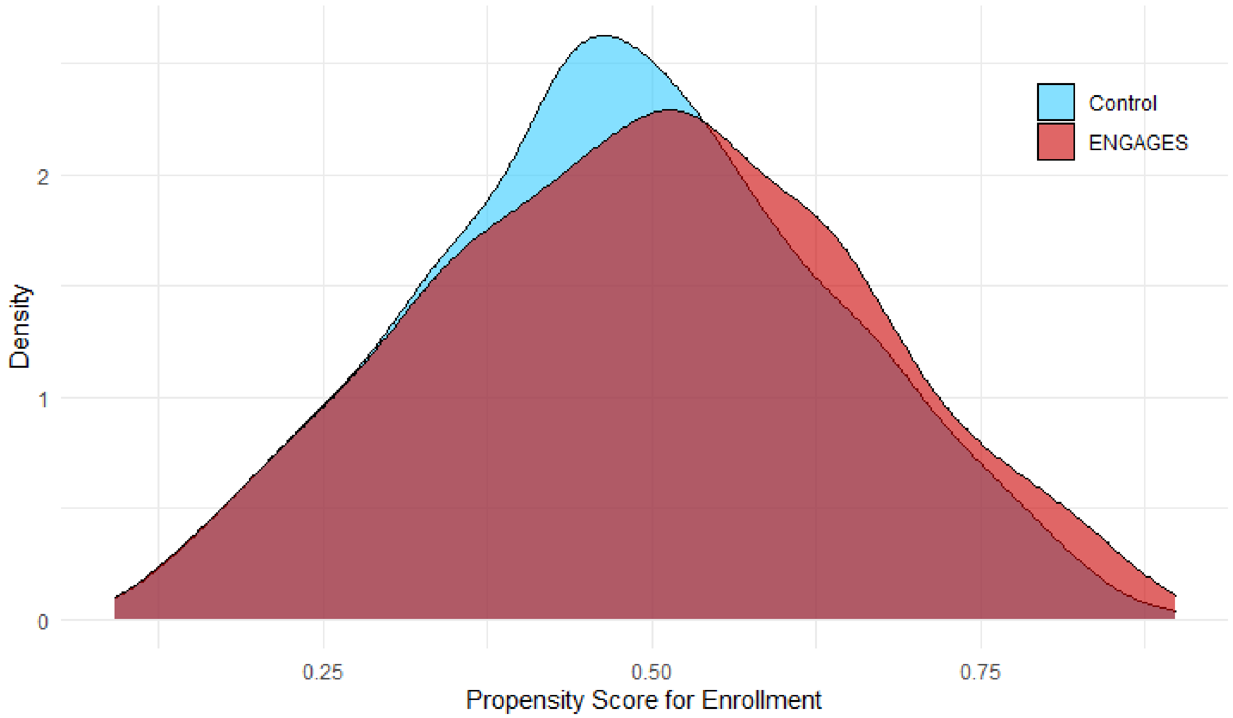

Density plot of propensity score for enrollment in the ENGAGES trial among matched patients in the control and intervention groups. The median pairwise difference in propensity scores was 0.016 (interquartile range 0.001 to 0.028).

© 2022 Fritz BA et al. *JAMA Network Open*.
