# Supplementary material for: Association of a Perioperative Multicomponent Fall Prevention Intervention With Falls and Quality of Life After Elective Inpatient Surgical Procedures
Source: JAMA Netw Open. 2022 Mar 11;5(3):e221938. doi: 10.1001/jamanetworkopen.2022.1938 (PMC8917421; doi:10.1001/jamanetworkopen.2022.1938)
Supplement: Supplement 2. — Nonauthor Collaborators [file jamanetwopen-e221938-s002.pdf]

Supplemental Online Content: Nonauthor Collaborators

*Indicates required information. Only first name, last name, and suffix will appear in PubMed.

## *Group Name(s): The ENGAGES Research Group

| ***First Name and Middle Initial(s)** | ***Last Name** | ***Suffix (eg, Jr, III)** | Academic Degrees | Institution | Location (city, state/province, country) | Role or Contribution, eg, chair, principal investigator | Group (if more than 1 Group listed in the byline) and/or Subgroup (eg, Steering Committee) |
|---|---|---|---|---|---|---|---|
| Ginika | Apakama | | MD | Washington University in St. Louis | St. Louis, MO, USA | Research Assistant | |
| Amrita | Aranake-Chrisinger | | MD | Washington University in St. Louis | St. Louis, MO, USA | Research Assistant | |
| Jacob | Bolzenius | | PhD | Washington University in St. Louis | St. Louis, MO, USA | Research Assistant | |
| Thaddeus P | Budelier | | MD, MSF | Washington University in St. Louis | St. Louis, MO, USA | Research Coordinator | |
| Jamila | Burton | | BS | Washington University in St. Louis | St. Louis, MO, USA | Research Assistant | |
| Victoria | Cui | | BS | Washington University in St. Louis | St. Louis, MO, USA | Research Assistant | |
| Daniel A | Emmert | | MD, PhD | Washington University in St. Louis | St. Louis, MO, USA | Investigator | |
| Shreya | Goswami | | MBBS, DNE | Washington University in St. Louis | St. Louis, MO, USA | Research Coordinator | |
| Thomas J | Graetz | | MD | Washington University in St. Louis | St. Louis, MO, USA | Investigator | |
| Shelly | Gupta | | BS | Washington University in St. Louis | St. Louis, MO, USA | Research Assistant | |
| Katherine | Jordan | | BS | Washington University in St. Louis | St. Louis, MO, USA | Research Assistant | |
| Hannah R | Maybrier | | BS | Washington University in St. Louis | St. Louis, MO, USA | Research Coordinator | |
| Sherry L | McKinnon | | BS | Washington University in St. Louis | St. Louis, MO, USA | Regulatory Specialist | |
| Angela M | Mickle | | MS | Washington University in St. Louis | St. Louis, MO, USA | Research Coordinator | |
| Maxwell R | Muench | | BS | Washington University in St. Louis | St. Louis, MO, USA | Research Assistant | |
| Matthew R | Murphy | | BS | Washington University in St. Louis | St. Louis, MO, USA | Research Assistant | |
| Jordan | Oberhaus | | BS | Washington University in St. Louis | St. Louis, MO, USA | Research Assistant | |
| Ben J | Palanca | | MD, PhD | Washington University in St. Louis | St. Louis, MO, USA | Investigator | |
| Daniel | Park | | BS | Washington University in St. Louis | St. Louis, MO, USA | Research Assistant | |
| Aamil | Patel | | BS | Washington University in St. Louis | St. Louis, MO, USA | Research Assistant | |
| James W | Spencer | | BS | Washington University in St. Louis | St. Louis, MO, USA | Research Assistant | |
| Tracey W | Stevens | | MD | Washington University in St. Louis | St. Louis, MO, USA | Investigator | |
| Patricia | Strutz | | BS | Washington University in St. Louis | St. Louis, MO, USA | Research Assistant | |
| Catherine M | Tedeschi | | BS | Washington University in St. Louis | St. Louis, MO, USA | Research Assistant | |
| Brian A | Torres | | DNP | Washington University in St. Louis | St. Louis, MO, USA | Investigator | |
| Emma R | Trammel | | BS | Washington University in St. Louis | St. Louis, MO, USA | Research Assistant | |
| Ravi T | Upadhyayula | | BS | Washington University in St. Louis | St. Louis, MO, USA | Research Assistant | |
| Anke C | Winter | | MD, MSc | Washington University in St. Louis | St. Louis, MO, USA | Investigator | |
| Nan | Lin | | PhD | Washington University in St. Louis | St. Louis, MO, USA | Statistician | |

Supplemental Online Content: Nonauthor Collaborators

*Indicates required information. Only first name, last name, and suffix will appear in PubMed.

| ***First Name and Middle Initial(s)** | ***Last Name** | ***Suffix (eg, Jr, III)** | Academic Degrees | Institution | Location (city, state/province, country) | Role or Contribution, eg, chair, principal investigator | Group (if more than 1 Group listed in the byline) and/or Subgroup (eg, Steering Committee) |
|---|---|---|---|---|---|---|---|
| Eric | Jacobsohn | | MBChB, MI | University of Manitoba | Winnipeg, Manitoba, Canad | Investigator | |
| Tamara | Fong | | MD, PhD | Beth Israel-Deaconess Medical Cente | Boston, MA, USA | Consultant | |
| Jackie | Gallagher | | MS | Beth Israel-Deaconess Medical Cente | Boston, MA, USA | Consultant | |
| Sharon K | Inouye | | MD, MPH | Beth Israel-Deaconess Medical Cente | Boston, MA, USA | Investigator | |
| Eva M | Schmitt | | PhD | Beth Israel-Deaconess Medical Cente | Boston, MA, USA | Investigator | |
| Spencer J | Melby | | MD | Washington University in St. Louis | St. Louis, MO, USA | Investigator | |
| Jennifer | Tappenden | | MFA | Washington University in St. Louis | St. Louis, MO, USA | Data Analyst | |
